# A novel Na_v_1.8-FLPo driver mouse for intersectional genetics to uncover the functional significance of primary sensory neuron diversity

**DOI:** 10.1016/j.isci.2024.109396

**Published:** 2024-03-05

**Authors:** Pascale Malapert, Guillaume Robert, Elena Brunet, Jean Chemin, Emmanuel Bourinet, Aziz Moqrich

**Affiliations:** 1 Aix-Marseille Université, CNRS, Institut de Biologie du Développement de Marseille, UMR 7288, case 907, 13288 Marseille Cedex 09, Marseille, France; 2 Institut de Génomique Fonctionnelle (IGF), Université de Montpellier, CNRS, INSERM, Montpellier, France

**Keywords:** Neuroscience, Molecular biology, Cellular biology

## Abstract

The recent development of single-cell and single-nucleus RNA sequencing has highlighted the extraordinary diversity of dorsal root ganglia neurons. However, the few available genetic tools limit our understanding of the functional significance of this heterogeneity. We generated a new mouse line expressing the flippase recombinase from the *scn10a* locus. By crossing Na_v_1.8^Ires−FLPo^ mice with the Advillin^Cre^ and RC::FL-hM3Dq mouse lines in an intersectional genetics approach, we were able to obtain somatodendritic expression of hM3Dq-mCherry selectively in the Na_v_1.8 lineage. The bath application of clozapine N-oxide triggered strong calcium responses selectively in mCherry^+^ neurons. The intraplantar injection of CNO caused robust flinching, shaking, and biting responses accompanied by strong cFos activation in the ipsilateral lumbar spinal cord. The Na_v_1.8^Ires−FLPo^ mouse model will be a valuable tool for extending our understanding of the *in vivo* functional specialization of neuronal subsets of the Na_v_1.8 lineage for which inducible Cre lines are available.

## Highlights

• Generation of a new mouse line expressing the flippase recombinase in Na_v_1.8 lineage
• CNO triggers strong calcium response in Na_v_1.8^Ires−FLPo^::Advillin^Cre^:: RC::FL-hM3Dq mice
• Intraplantar injection of CNO causes robust nocifensive responses and strong spinal cFos

## Graphical abstract

**Figure:**
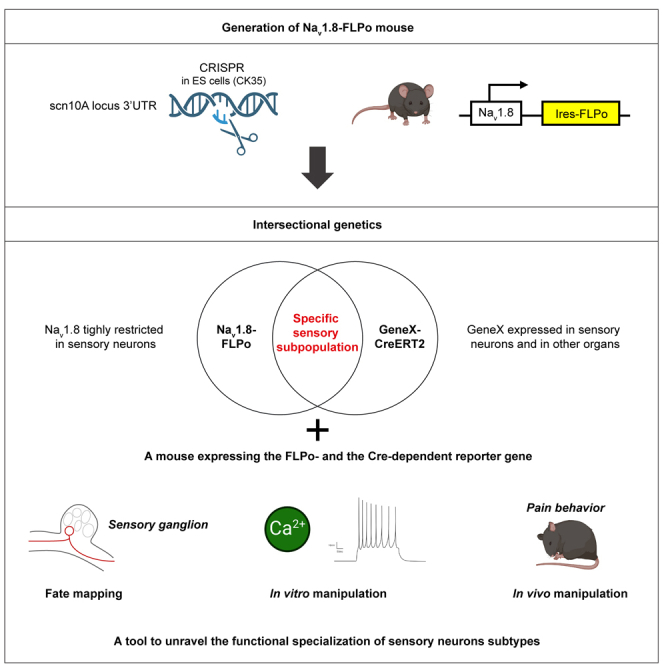

## Introduction

Primary somatosensory neurons are a highly diverse group of neurons that enable us to perceive and discriminate between diverse types of sensations from the outside world and the internal state of the body. The cell bodies of somatosensory neurons reside in sensory ganglia, including the dorsal root ganglia (DRG), trigeminal ganglia (TG), and other cranial nerve ganglia.^1^^,^^2^^,^^3^^,^^4^^,^^5^^,^^6^ Decades of research, including the recent development of single-cell RNA sequencing, have greatly increased our knowledge of the extraordinary diversity of DRG neurons. These neurons can be distinguished on the basis of cell body size, axon caliber and degree of myelination, peripheral and central projection patterns, and transcriptional profiles.^7^^,^^8^^,^^9^^,^^10^^,^^11^^,^^12^^,^^13^^,^^14^^,^^15^^,^^16^ However, despite this wealth of information, our understanding of the functional specialization of somatosensory neurons remains far from complete because of the lack of appropriate genetic tools selectively targeting these neuronal subsets with a very high degree of precision.

Knockout mice are the tools of choice for studying the functional role of a given gene in a particular population of neurons *in vivo.*^17^^,^^18^^,^^19^^,^^20^^,^^21^^,^^22^ However, interpretation of the results is often complicated by the transient or dynamic developmental expression of the gene of interest in the somatosensory system and its expression outside the somatosensory system. New genetic approaches based on the Cre-loxP system have been developed to overcome these problems.^23^^,^^24^^,^^25^^,^^26^^,^^27^^,^^28^^,^^29^ The use of such systems has led to the generation of a large number of mouse lines in which the Cre-recombinase drives gene inactivation or the targeted expression of effector molecules, such as fluorescent markers, calcium reporters, optogenetic actuators, or exogenous ligand-responsive receptors (designer receptors exclusively activated by designer drugs [DREADDs]) in particular subsets of somatosensory and dorsal horn spinal neurons.^4^^,^^30^^,^^31^^,^^32^^,^^33^^,^^34^^,^^35^^,^^36^^,^^37^ Despite these advances in genome-editing techniques, it is becoming increasingly clear that the recombinase-mediated expression of a single gene cannot provide the necessary resolution for studies of the functional specialization of a single cell type. The need to overcome this obstacle led to the development of intersectional genetic tools, pioneered by Qiufu Ma and Martyn Goulding, to restrict the effect of Cre lines specifically to spinal cord neurons.^37^^,^^38^^,^^39^^,^^40^^,^^41^^,^^42^^,^^43^^,^^44^ Intersectional genetics increases resolution through the use of a dual-recombinase system based on Cre and flippase (FLPo) to activate a conditional effector allele only in cells in which both recombinases are expressed. However, its use in the somatosensory system is hampered by the lack of mouse lines expressing the FLPo recombinase selectively in sensory neurons. In this study, we generated an FLPo-driver mouse line that expresses the FLPo recombinase from the 3′ UTR of the *Na*_*v*_*1.8* locus. This approach ensures that the Na_v_1.8 voltage-gated sodium channel remains intact and allows functional and selective expression of the FLPo recombinase in the Na_v_1.8 lineage in DRG, TG, and jugular nodose ganglia (JNG) neurons. Crossing of Na_v_1.8^Ires−FLPo^ with Advillin-^Cre^ and RC::FL-hM3Dq reporter mice resulted in robust expression of the excitatory DREADD in a large number of DRG neurons. Bath applications of clozapine N-oxide (CNO) triggered strong calcium responses selectively in the intersecting DRG neurons in which both recombinases were expressed, demonstrating induction of the canonical Gq pathway and subsequent neuronal activation. Intraplantar injections of CNO caused robust flinching, shaking, and biting responses accompanied by strong cFos activation in the ipsilateral lumbar spinal cord.

## Results

### Generation of Na_v_1.8^Ires−FLPo^ mice

**Figure 1:**
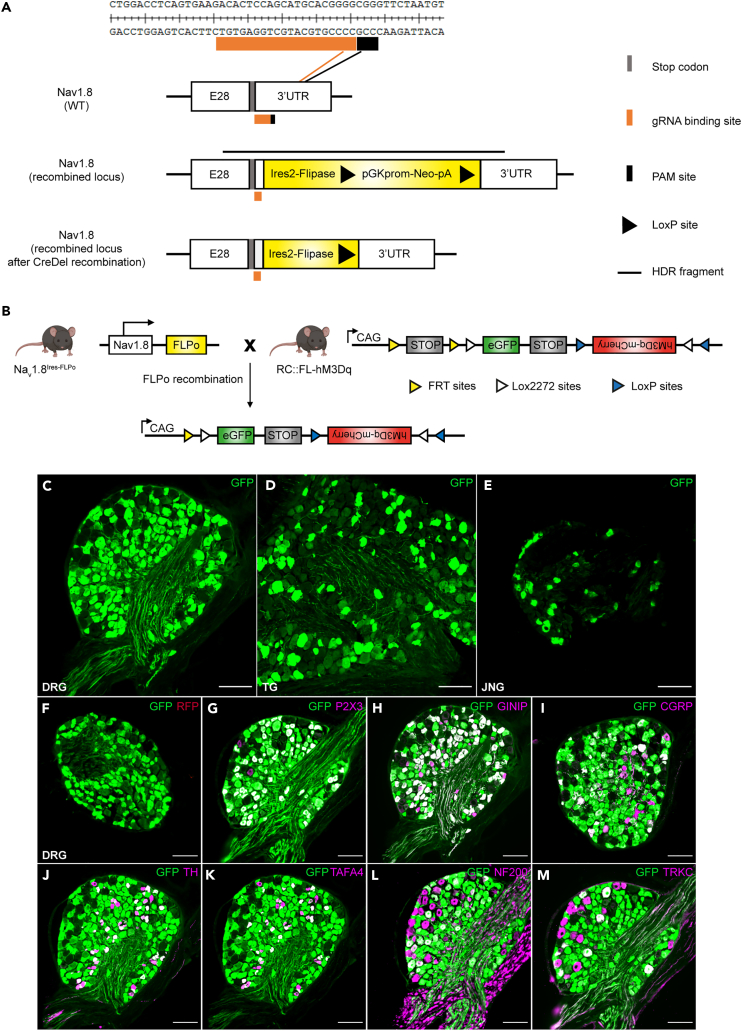
Generation of the Na_v_1.8^Ires−FLPo^ mouse model with functional and selective Flippase expression in sensory neurons of the Na_v_1.8 lineage (A) Schematic representation of *scn10a* locus gene editing. CRISPR-Cas9 technology was used to insert an Ires-FLPo cassette was downstream of exon28 (E28) immediately after the stop codon. (B) Schematic representation of the strategy for mating between a Na_v_1.8^Ires−FLPo^ mouse and a reporter RC::FL-hM3Dq mouse. FLPo recombination in the Na_v_1.8^Ires−FLPo/+^:RC::FL-hM3Dq mice leads to excision of the FRT-flanked transcriptional STOP cassette, resulting in EGFP expression in the Na_v_1.8 lineage. (C–E) GFP immunostaining on thoracic DRG (C), trigeminal ganglia (TG) (D), and jugular-nodose-ganglia (JNG) sections (E) from a Na_v_1.8^FLPo/+^:RC-FL-hM3Dq mouse. (F) Thoracic DRG section co-immunolabeled for GFP (green) and RFP (red). (G–M) Thoracic DRG sections co-immunolabeled for GFP (green) and P2X3 (G), GINIP (H), CGRP (I), TH (J), TAFA4 (K), NF200 (L), or TRKC (M) (magenta) (n = 2 mice). Scale bar: 100 μm; n = 2 mice.

As expression of the tetrodotoxin (TTX)-resistant Na_v_1.8 sodium channel is tightly restricted to somatosensory neurons, we sought to generate a new mouse model expressing the FLPo recombinase rather than the Cre recombinase in the Na_v_1.8 lineage. Given the important physiological role of this channel in sensory neurons, we used CRISPR technology to knockin an Ires-FLPo cassette at the 3′ UTR of the *Scn10a* locus (Figure 1A). With the design used, the resulting allele should produce a bicistronic mRNA encoding a functional Na_v_1.8 channel and the FLPo recombinase in the Na_v_1.8 lineage. For validation of our mouse model, we first crossed Na_v_1.8^Ires−FLPo^ mice with RC::FL-hM3Dq mice^45^ (Figure 1B). In the progeny of this cross, the FLPo excised the FRT-flanked transcription STOP cassette, allowing the expression of EGFP in a large number of DRG, TG, and JNG neurons (Figures 1C–1E; S1D for quantification). To determine the extent to which EGFP expression overlapped with the distribution of *Na*_*v*_*1.8* mRNA, we performed *in situ* hybridization for Na_v_1.8 followed by immunostaining for EFGP (Figure S1A). We found that 85.2% ± 0.6% of EGFP^+^ neurons expressed *Na*_*v*_*1.8* mRNA, and 85.4% ± 2.1% of *Na*_*v*_*1.8*^*+*^ neurons expressed EGFP (Figures S1A and S1B). We were, thus, able to capture both persistent and transient *Na*_*v*_*1.8*-expressing neurons, but the IRES-mediated translation of FLPo failed to trigger the excision of the FRT-flanked STOP cassette in a small subset of DRG neurons. Consistent with these findings, 82.9% ± 0.4% of neurons were EGFP^+^ in homozygous Na_v_1.8^Ires−FLPo/Ires−FLPo^:: RC::FL-hM3Dq mice, versus 71.0% ± 1.9% in heterozygous Na_v_1.8^Ires−FLPo/+^:: RC::FL-hM3Dq mice (Figures S1C and S1D).

Given the major functional role of the Na_v_1.8 channel in sensory neurons, we checked that our genetic approach did not interfere with the function of this channel. We performed electrophysiological recordings of sodium currents in GFP^+^ DRG neurons obtained from heterozygous Na_v_1.8^Ires−FLPo/+^::RC::FL-hM3Dq and homozygous Na_v_1.8^Ires−FLPo/Ires−FLPo^::RC::FL-hM3Dq mice. We focused on putative Na_v_1.8 currents by performing recordings at a holding potential of −40 mV, in the presence of 300 nM TTX, 0.1 mM external calcium, and 0.1 mM cadmium to inhibit voltage-gated calcium channels. Under these conditions, a 30 ms test pulse from −40 mV to +50 mV triggered robust TTX-resistant sodium currents, the maximal current being recorded at 0 mV (Figure S1E). Importantly, there was no difference in maximal current density between the two genotypes (Figure S1F), demonstrating that our targeting strategy allowed the expression of a functional Na_v_1.8 channel in both heterozygous and homozygous mice.

We then performed a series of double-immunohistochemistry (IHC) experiments on DRG sections. Using anti-GFP and anti-RFP antibodies, we demonstrated the high-fidelity FLPo-mediated expression of EGFP, as no expression of mCherry was observed in DRG neurons (Figure 1F). We also found that FLPo-mediated recombination targeted C-fibers and Aδ nociceptors expressing P2X3, GINIP, CGRP, TH, and TAFA4 (Figures 1G–1K). Our targeting strategy also captured a large number of large-diameter Aβ neurons expressing NF200 and TrkC (Figures 1L and 1M).

**Figure 2:**
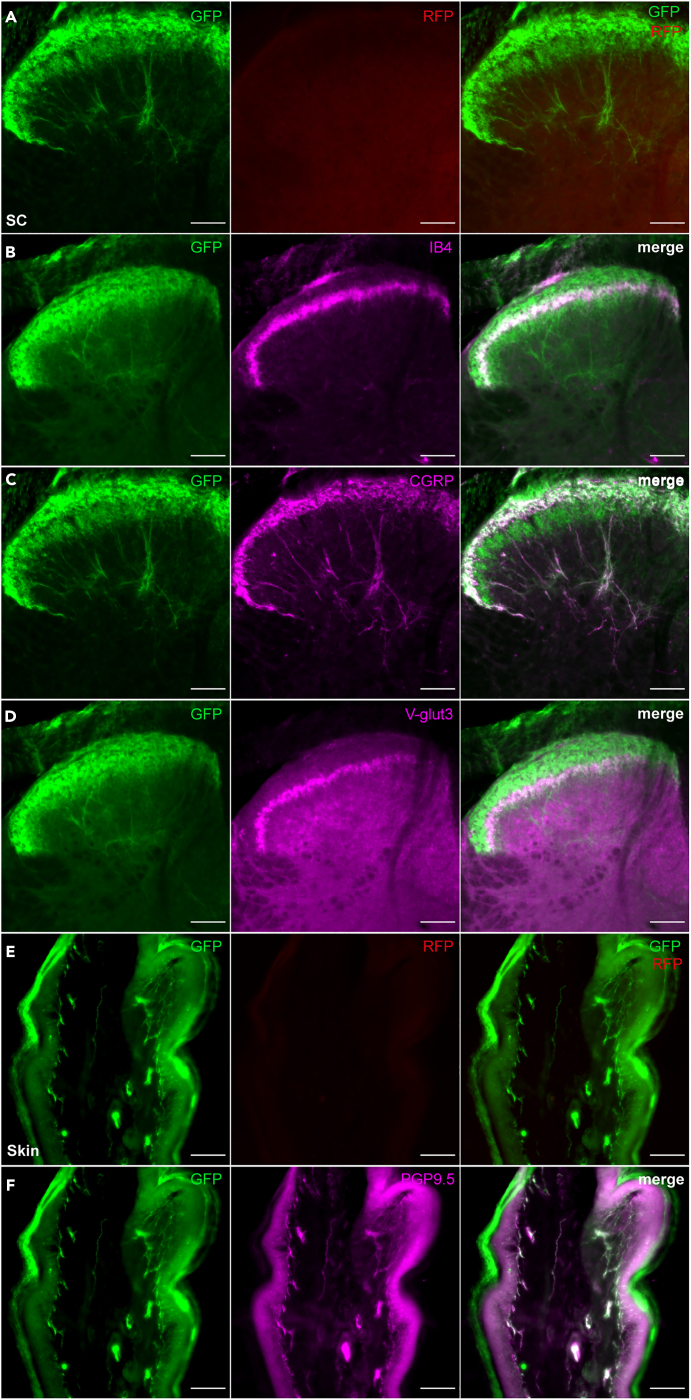
Mapping of central and peripheral skin projections of sensory neurons of the Na_v_1.8 lineage (A) L3 to L5 lumbar segments dorsal horn of the spinal cord (LDHSC) from Na_v_1.8^Ires−FLPo/+^:RC::FL-hM3Dq mouse co-immunolabeled for GFP (green) and RFP (red). (B–D) LDHSC co-immunolabeled for GFP (green) and IB4 (B), CGRP (C), or VGLUT3 (D) (magenta). (E) Hind-paw glabrous skin section co-immunolabeled for GFP (green) and RFP (red). (F) Hind-paw glabrous skin section co-immunolabeled for GFP (green) and PGP9.5 (magenta). Scale bar: 100 μm, n = 2 mice.

For identification of the central and peripheral projections of EGFP^+^ DRG neurons, we performed double-IHC on sections from the lumbar segment of the spinal cord and the skin. Consistent with the expression of EGFP predominantly in C- and Aδ fibers, EGFP^+^ central terminals were highly restricted to the dorsal horn of the spinal cord, particularly in laminae I (labeled with CGRP) and laminae II (labeled with IB4 and VGLUT3) (Figures 2B–2D). At the periphery, massive EGFP innervation of the skin was visualized through the colabeling of EGFP^+^ nerve endings with the pan-neuronal marker PGP9.5 (Figure 2F). No RFP staining was observed in the central and peripheral endings of DRG neurons, further demonstrating an absence of leakage for the cassette expressing the -hM3Dq-mCherry fusion protein (Figures 2A and 2E). EGFP^+^ terminals were also observed in several visceral organs, including the bladder (Figure S2A) and jejunum (Figure S2B), but not the liver (Figure S2C). Outside the primary sensory nervous system, our genetic design captured very strong expression of the Na_v_*1.8* allele in the cardiac conduction system (Figure S2D) and in a few EGFP^+^/PV^−^ neurons in the limbic system (Figures S2E and S2F). Together, these data show that the mouse model generated drives FLPo recombinase activity with very high fidelity in the Na_v_1.8 lineage and highlights several hotspots of *Na*_*v*_*1.8* gene activity in other organs, including the brain and heart.

### The Na_v_1.8^Ires−FLPo^ mouse is suitable for use in intersectional genetics

**Figure 3:**
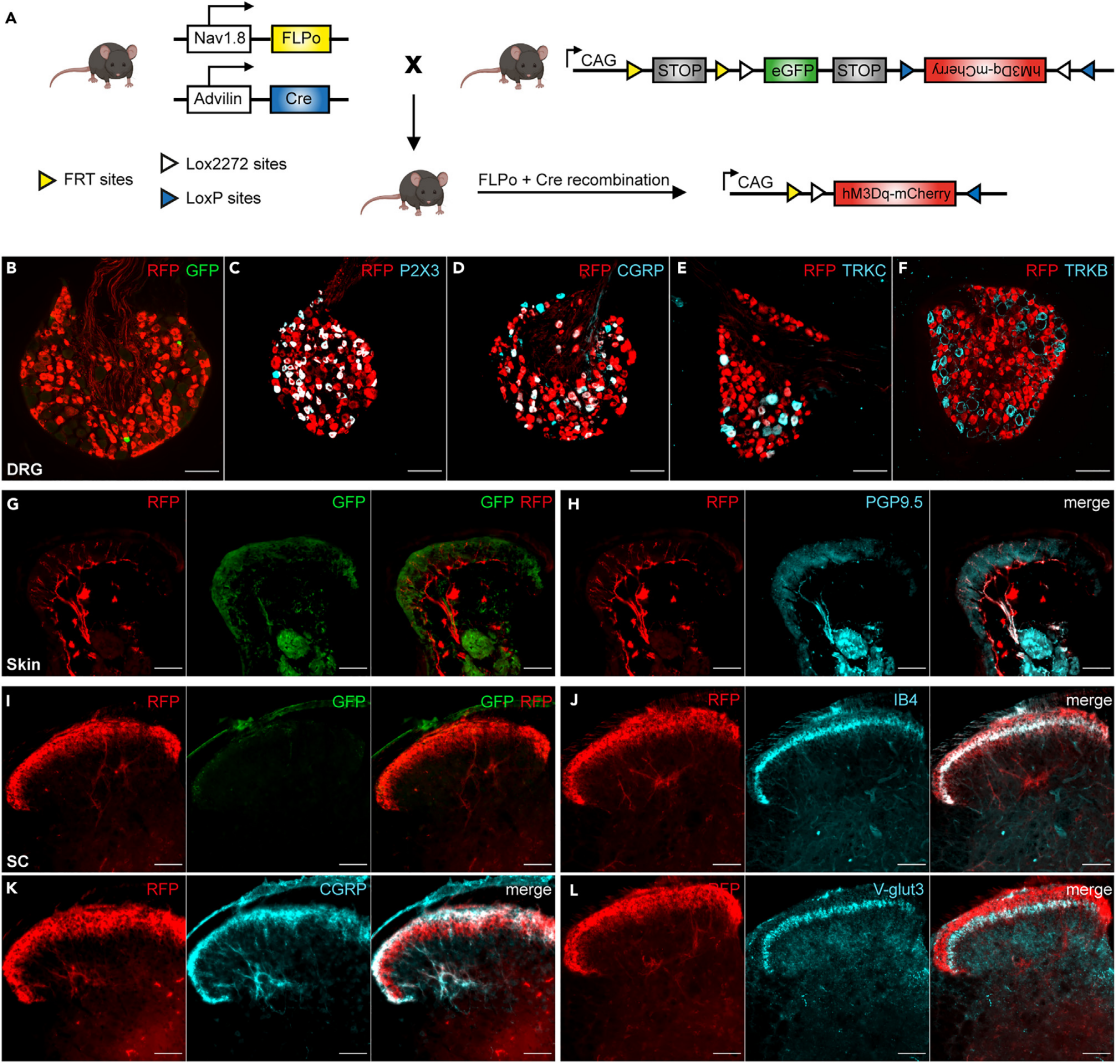
Na_v_1.8^Ires−FLPo^ mice are suitable for use in intersectional genetics (A) Schematic representation of the strategy for mating between a Na_v_1.8^Ires−FLPo/+^::Advillin^Cre^ mouse and a reporter RC::FL-hM3Dq mouse. This strategy allows the concomitant excision of the EGFP cassette and inversion of the hM3Dq cassette toward the right reading frame, leading to production of the hM3Dq-mCherry fusion protein. (B) Thoracic DRG section from a Na_v_1.8^Flpo/+^::Advillin^Cre/+^:: RC::FL-hM3Dq mouse co-immunolabeled with RFP (red) and GFP (green). (C–F) Thoracic DRG sections co-immunolabeled for RFP (red) and P2X3 (C), CGRP (D), TrkC (E), or TrkB (F) (cyan). (G) Hind-paw glabrous skin section co-immunolabeled for RFP (red) and GFP (green). (H) Hind-paw glabrous skin section co-immunolabeled for RFP (red) and PGP9.5 (cyan). (I) Dorsal horn of the spinal cord (DHSC) co-immunolabeled for RFP (red) and GFP (green). (J–L) LDHSC co-immunolabeled for RFP (red) and IB4 (J), CGRP (K), or VGLUT3 (L). Scale bar: 100 μm; n = 2 mice.

We assessed the suitability of our newly generated mouse model for use in intersectional genetics by crossing Na_v_1.8^Ires−FLPo^::RC::FL-hM3Dq mice with Advillin-Cre (Adv^−Cre^) mice.^46^ In the triple-transgenic offspring, further exposure to Cre triggered the excision of EGFP and inverted the cassette mediating somatodendritic expression of the hM3Dq/mCherry fusion protein (Figure 3A). Indeed, double-IHC with antibodies against GFP and RFP showed that all DRG neurons expressed mCherry rather than EGFP (Figure 3 B). The double-recombination event occurred selectively in Na_v_1.8 lineage because mCherry was co-expressed with markers of C-fibers and Aδ nociceptive neurons labeled with P2X3 and CGRP (Figures 3C and 3D) and was excluded from large-diameter neurons expressing TrkC and TrkB (Figures 3E and 3F). Consistent with this finding, mCherry^+^ terminals no longer expressed EGFP peripherally (Figure 3G) or centrally (Figure 3I), and they densely innervated the skin as free nerve endings, peripherally (Figure 3H), and were restricted to laminae I and II of the dorsal horn of the spinal cord labeled with IB4, CGRP, and VGLUT3 (Figures 3J–3L).

### CNO induced strong activation of the Na_v_1.8 lineage both *in vitro* and *in vivo*

**Figure 4:**
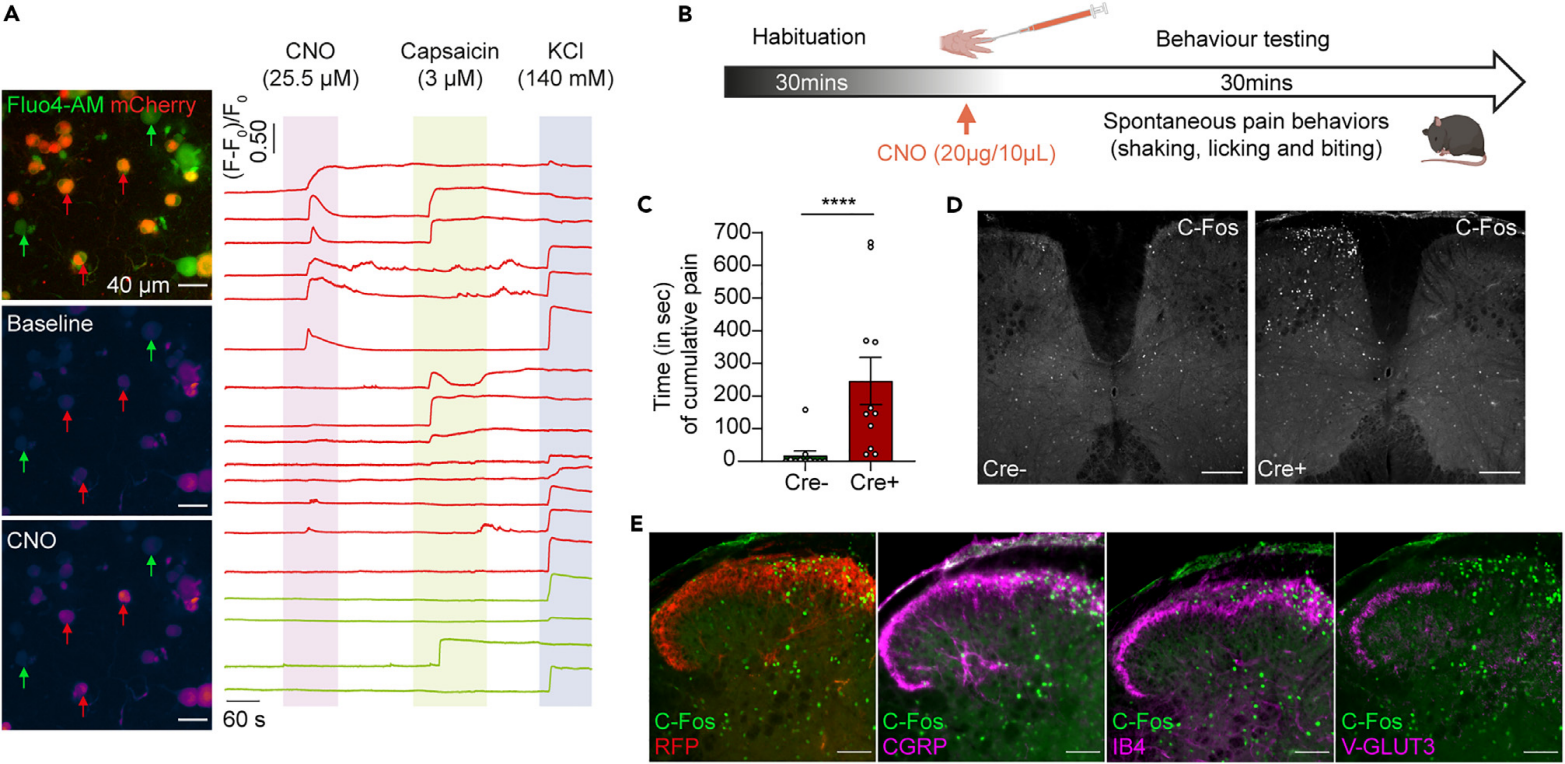
Manipulation *in vitro* and *in vivo* of the Na_v_1.8-lineage sensory neurons (A) Representative images highlighting mCherry-positive (red arrows) and -negative (green arrow) DRG neurons before and after CNO application (left). Calcium responses of selected mCherry-positive (red traces) and -negative (green traces) neurons to CNO, capsaicin (Cap), and KCl (right). The data were obtained from two different mice and six different ibidi plates. The data were obtained from two different mice. In total, the experiment was repeated 3 times in 3 different culture dishes. 68 neurons were analyzed (52 m-Cherry^+^ neurons and 12 m-cherry^-^ neurons). 59.61% of m-Cherry^+^ neurons responded to CNO, 46.15% to capsaicin, and 61.54% to KCl. None of the m-Cherry^-^ neurons responded to CNO, 4% of the neurons responded to capsaicin, and 75% to KCl. (B) Illustration of the behavioral design before and after the intraplantar injection of CNO into control Na_v_1.8^Ires−FLPo/+^:Advillin^+/+^::RC::FL-hM3Dq and experimental Na_v_1.8^Ires−FLPo/+^::Advillin^Cre/+^:: RC::FL-hM3Dq mice. (C) Cumulative duration of the spontaneous pain behavior (in seconds) recorded in Cre^−^ (n = 11) and Cre^+^ (n = 11) mice. Data are presented as the mean ± SEM (unpaired Mann-Whitney test, ∗∗∗∗p < 0.0001). (D) C-cFos immunostaining on spinal cord (SC) sections from Cre^−^ (n = 3) and Cre^+^ (n = 3) mice, 1 h after CNO injection. (E) Ipsilateral side of the lumbar dorsal horn of the spinal cord from a Cre^+^ mouse, co-immunolabeled for cFos (green) and RFP (n = 4, red) and for -cFos (green) and CGRP (n = 2), IB4 (n = 4), or VGLUT3 (n = 4) (magenta). Scale bar: 200 μm (D), 100 μm (E).

Given that mCherry is fused to hM3Dq, the data suggest that the excitatory DREADD is expressed in the cell bodies in addition to the central and peripheral terminals of the Na_v_1.8 lineage. CNO administration was therefore expected to induce the canonical G_q_ pathway, leading to depolarization/activation of the targeted neurons. We tested this hypothesis by performing calcium imaging on cultured mCherry-positive and -negative neurons (Figure 4A left). We monitored hM3Dq-induced calcium responses to 25.5 μM CNO, followed by TRPV1-induced responses to 3 μM capsaicin (Figure 4A right). CNO triggered calcium responses in 31 of 52 mCherry^+^ neurons, half of which also responded to capsaicin. By contrast, none of the mCherry^−^ neurons responded to CNO. The overall activity of all neurons was assessed by monitoring their calcium responses in the presence of 140 mM KCl.

We then investigated the effect of CNO administration *in vivo*. We used two different readouts, one behavioral and the other molecular. Intraplantar injections of CNO triggered robust flinching, biting, and shaking responses in the injected paw (Figures 4B and 4C and Supplemental movies). This very pronounced behavior began immediately after CNO injection and lasted 30 min. This phenotype was selective, observed only in Cre^+^ mice, with Cre^−^ mice not displaying such behavior following CNO injection. As we also wanted to have a molecular readout of the effects of CNO injection, mice were killed 1 h after CNO administration, and sections of the lumbar segments of the spinal cord were prepared and subjected to double-IHC with anti-cFos antibody together with antibodies against CGRP, IB4, or VGLUT3. Very strong nuclear cFos immunostaining was observed selectively in the ipsilateral dorsal horn of the spinal cord in Cre^+^ mice but not in Cre^−^ mice (Figure 4D). Interestingly, cFos immunostaining was concentrated in the mediolateral part of the spinal cord, which is known to be innervated by lumbar DRG neurons projecting into the glabrous skin of the hind paw (Figure 4E). This spinal region is innervated by CGRP^+^ and IB4^+^ central terminals but not by those labeled with VGLUT3, a specific marker of C-low threshold mechanoreceptors, which innervate exclusively hairy skin (Figure 4E).

## Discussion

In this study, we generated a new mouse model that will be extremely useful for use in elegant intersectional genetics approaches in mice.^38^ Such approaches make it possible to achieve a high degree of specificity in investigations of the biological functions of a specific subset of cells, identified by the expression of a particular gene, even if this gene of interest is also expressed in tissues other than the tissue of interest. Single-cell and single-nucleus RNA sequencing techniques have made it possible to identify a wide range of molecularly defined subpopulations of sensory neurons in the somatic sensory nervous system,^47^ consistent with the broad range of sensory modalities that can be perceived by our bodies. Efforts to decipher the precise functional specialization of each of these neuronal subsets have been hampered by a lack of appropriate genetic tools for the selective and specific targeting of particular subsets. The mouse model developed here will facilitate such studies, through combination with appropriate mouse Cre driver lines. We show here that crossing the Na_v_1.8^Ires−FLPo^, the Advillin^Cre^, and RC::FL-hM3Dq reporter mice allows a the expression of a putatively functional excitatory DREADD throughout the entire Na_v_1.8 lineage, as demonstrated by the expression of the mCherry. Using calcium imaging, we showed that 60% of the intersected neurons responded to bath applications of CNO, demonstrating that only a proportion of mCherry-expressing DRG neurons were competent to respond to bath application of CNO. However, from a behavioral point of view, a functional excitatory DREADD was fully validated *in vivo* as intraplantar injections of CNO triggered robust nocifensive behavior accompanied by strong cFos activation in the mediolateral part of the spinal dorsal horn, the spinal region in which the neurons innervating the glabrous skin of the hind paw terminate. Our results demonstrate that this new mouse model is a highly suitable tool for deciphering the functional specialization of a wide range of primary sensory neurons subsets by intersectional genetics.

Over the last two decades, the sensory biology community has generated a large number of mouse lines in which Cre-recombinase is expressed under the control of promoters of genes selectively expressed in particular subsets of DRG neurons. Unfortunately, many of these mouse lines lack temporal control over Cre recombination events, particularly for genes displaying dynamic expression during embryonic development and/or at postnatal stages. As a result, it is often not only the DRG neurons expressing the gene of interest that ends up being targeting, but also its lineage. For example, the NGF receptor TrkA is expressed in 90% of DRG neurons during development and at birth.^48^ During the first 3 weeks of postnatal life, *trkA* expression is switched off in more than half the TrkA-expressing neurons, which instead express the glial cell-derived neurotrophic factor receptor Ret.^49^ Therefore, in the adult DRG, *trkA* ends up being expressed in less than 30% of all adult DRG neurons. Likewise, MRGPRB4^+^ neurons account for fewer than half the neurons fate-mapped with the mrgprb4^Cre^ mouse.^31^ Thus, targeting the lineages in which these genes are expressed does not provide any information about the functional specialization of the subsets of neurons actually expressing TrkA or MRGPRB4 in the adult mouse.

More mouse lines expressing inducible Cre will need to be generated to overcome these issues. Indeed, crossing the Na_v_1.8^Ires−FLPo^ mouse with a mouse that expresses an inducible Cre in a small subset of cells of the Na_v_1.8 lineage makes it possible to express effector molecules in a highly controlled manner. With such approaches, the subset of neurons of interest can be genetically marked, activated, inactivated, or ablated, at will. These manipulations can be achieved *in vitro* or *in vivo*, under physiological or pathological conditions. The high degree of spatial and temporal specificity is the key advantage of such systems. The Na_v_1.8^Ires−FLPo^ mouse is an excellent model as it spatially restricts FLPo expression to a very small number of tissues, such as the DRG, TG, nodose ganglia (NG), and the conduction tissue of the heart. Temporal control over the expression and activation of the effector molecules depends on the induction of Cre recombination.

Na_v_1.8^Ires−FLPo^ restricts FLPo recombination to a small number of tissues, but there will inevitably be some circumstances in which a gene of interest is expressed in a subset of the Na_v_1.8 DRG lineage, in the NG for example. This dual expression is necessarily problematic as manipulating the activity of NG neurons at the same time as that of DRG neurons may complicate interpretation of the results. It is possible to overcome this problem by using viral infection to express the dual-recombinase-dependent effector molecules in organs targeted by DRG but not by NG neurons. This strategy was recently used in an elegant study by the Ginty laboratory for selective visualization of the projection pattern and manipulation of the activity of two different somatosensory neuron subtypes innervating the Krause corpuscles of the mouse penis and clitoris.^36^

Finally, the power of our approach lies in the fact that our mouse model allows expression of the FLPo recombinase without affecting the integrity of the *scn10a* locus, as it expresses the wild-type version of the Na_v_1.8 channel. Our model, which does not impair the very important function of this channel in sensory neurons, can, therefore, be used in a wide range of intersectional genetic approaches with no potential confounding effects due to the absence of one copy of the Na_v_1.8 allele.

### Limitations of the study

In the present study, we generated and validated a new mouse model expressing the FLPo from the *scn10a* (Na_v_1.8) locus. Using the IRES cassette to drive the translation of the FLPo does not target 100% of Na_v_1.8 lineage. Therefore, depending on the subsets of DRG neurons to be targeted, one will need to use homozygous mice.

The second limitation of the study is related to the calcium imaging experiments. Only two mice and six plates were used to generate the data presented in Figure 4A. This part of the study was mainly performed to show that our newly generated mouse model allows the expression of a functional excitatory DREADD. It turns out that this experiment shows that CNO triggers calcium responses in only a fraction of DRG neurons, demonstrating that not all DRG neurons are competent to respond to CNO in the calcium imaging experiments.

## STAR★Methods

### Key resources table

**Table:** 

REAGENT or RESOURCE	SOURCE	IDENTIFIER
**Antibodies**		

rabbit anti-cFos	Millipore	cat# ABE457; RRID:AB_2631318
chicken anti-GFP	Thermofisher	cat# A10262; RRID:AB_2534023
rat anti-RFP	ChromoTek	cat# 5f8; RRID: AB_2336064
rabbit anti-P2X3	Neuromics	cat# RA10109; RRID:AB_2157931
rat anti-Ginip	Gaillard et al. 2014	N/A
rabbit anti-CGRP	Calbiochem	cat# PC205L
rabbit anti-TH	Sigma	cat# AB152
rat anti-Tafa4	Gift from Sophie Ugolini-CIML	N/A
rabbit anti-neurofilament	Sigma	cat# AB1987
goat anti-TrkC	R&D Systems	cat# AF1404; RRID:AB_2155412
goat anti-CGRP	OriGene	cat# BP022
guinea pig anti-vGlut3	Synaptic System	cat# 135204
rabbit anti PGP9.5	Thermofisher	cat# PA5-29012; RRID:AB_2546488
horseradish peroxidase anti-digoxigenin antibody	Roche	Cat#11207733910; RRID:AB_514500

**Chemicals, peptides, and recombinant proteins**		

Clozapine N-Oxide	Tocris	cat# 6329
PBS	Eurobio	cat# CS0PBS01-08
Paraformaldehyde	Electron Microscopy Sciences	cat# 15714
Sucrose	Carl Roth	cat# 4621.1
OCT medium	Leica Microsystèmes SAS	cat# 14020108926
Agarose	Carl Roth	cat# 2267.4
Penicillin/Streptomycine	Life Technologies	cat# 15140-122
Percoll	Dutscher	cat# 17-0891-01
Capsaicin	Sigma-Aldrich	cat# M2028-50MG
Fluo4-AM	Thermofisher Scientific	cat# F-14201
DIG RNA Labeling Mix, 10x	Roche	cat# 11277073910
TSA-Cy3	Perkin Elmer Life Sciences	cat# FP1170
Phusion Hign Fidelity DNA polymerase	Thermofisher Scientific	cat# F-530S
AatII restriction enzyme	New England Biolabs	cat# R0117S
BbsI restrtiction enzyme	New England Biolabs	cat# R3539S
SacI restriction enzyme	New England Biolabs	cat# R3156S
Collagenase II	Life Technologies	cat# 17101015
Dispase	Life Technologies	cat# 17105041
HBSS media	Life Technologies	cat# 14025092
Leibovitz-15 medium	Life Technologies	cat# 11415049
Fœtal calf serum	Life Technologies	cat# 10270-106
Neurobasal medium	Life Technologies	cat# 10888022
Neurotrophin-4	Peprotech	cat# 450-03
Glial cell-derived neurotrophic factor	Life Technologies	cat# PHC7044
Poly-D-Lysine	Sigma-Aldrich	cat# A-003-E
Laminin	Sigma-Aldrich	cat# L2020-1MG
Donkey serum	Sigma-Aldrich	cat# D9663
Bovine serum albumine	Sigma-Aldrich	cat# A9418
OptiMEM medium	Life Technologies	cat# 31985070
Guide-it sgRNA screening system	Takara	cat# 632639

**Critical commercial assays**		

Nucleospin Gel and PCR cleanup kit	Macherey Nagel	cat# 740609
Nucleospin Plasmid mini kit	Macherey Nagel	cat# 740588
Megashortscript T7 transcription kit	Life Technologies	cat# AM1354
Megaclear kit	Life Technologies	cat# AM1908
EnGen mutation detection kit	New England Biolabs	cat# E3321
Nucleobond Xtra Midi Endofree kit	Macherey Nagel	cat# 740420
InFusion cloning kit	Takara	cat# 639678

**Experimental models: Organisms/strains**		

Advillin Cre	gift or Dr Fan Wang (Duke University)	RRID:IMSR_JAX:032536
RC::FL-hM3Dq	gift or Dr Patricia Jensen (NIEHS)	RRID:IMSR_JAX:026942
Na_v_1.8^Ires-FLPo^	generated in this study	N/A
C57BL/6J	Charles River Laboratories	N/A

**Oligonucleotides**		

Nav1.8-F1: ACACACTCTCTGGCCCTCTTAG	This paper	N/A
Nav1.8-R1: CCCTATGCAACTTCTCCAAGAT	This paper	N/A
Nav1.8-F2: TGGACATCCTCTTTGCCTTC	This paper	N/A
Nav1.8-R2-T7: TAATACGACTCACTATA GGGACCACCAGAAATGTCCTTGC	This paper	N/A
WT-F: CAAATGACAACGGTGGGCTC	This paper	N/A
WT-R: TAATGGCCGACCCTCAGGTA	This paper	N/A
Mut-R (in Ires cassette): CAAAGGGTCGCTACAGACGTT	This paper	N/A

**Recombinant DNA**		

pSpCas9(BB)-2A-Puro (PX459) V2.0 vector	gift from Feng Zhang	Addgene plasmid #62988

**Software and algorithms**		

Fiji	N/A	RRID: SCR_002285
Max-Chelator software	N/A	N/A
GraphPad Prism	La Jolla, CA	https://www.graphpad.com/
Pylon viewer Software	N/A	N/A
pCLAMP9	Molecular Devices	N/A
BioRender.com	N/A	N/A

**Other**		

Cryostat	Leica	cat#3050S
Superfrost plus slides	Epredia	cat# J7800AMNZ
Vibratome	Leica	cat# VT1200S
AxioImager	Zeiss	cat# M2

### Resource availability

#### Lead contact

Further information and requests for resources related to this study should be directed to, and will be fulfilled by, the lead contact Aziz Moqrich (aziz.moqrich@univ-amu.fr)

#### Materials availability

The newly generated mouse model will be available to scientific community upon request.

#### Data and code availability

• All software used are listed in the key resources table. This study did not generate any unique datasets or new code
• All data reported in this paper will be shared by the lead contact upon reasonable request
• Any additional information required to reanalyze the data reported in this paper is available from the lead contact upon request

### Experimental model and study participant details

#### Mice

Mice were maintained under standard housing conditions (22°C, 40% humidity, 12 h-light cycles, and free access to food and water). Special efforts were made to minimize the number of mice used in this study. All experiments were conducted on 8- to 12-week-old adult mice in accordance with European guidelines for the care and use of laboratory animals (Council Directive 86/609/EEC). All experimental procedures were approved by an independent ethics committee for animal experimentation, as required by the French law and in accordance with the relevant institutional regulations of the French legislation on animal experimentation, under license numbers 2015070217242262-V5#1537, and 34501-2022010309527657-v5. All experiments were performed in accordance with the ARRIVE guidelines.

AdvillinCre and RC::FL-hM3Dq mice were generously provided by Dr Fan Wang (Duke University) and Dr Patricia Jensen (National Institute of Environmental Health Sciences (NIEHS), respectively. The Na_v_1.8^Ires-FLPo^ mice were generated in this study.

### Method details

#### Generation of the Na_v_1.8^Ires-FLPo^ mouse line

##### Design of the CRISPR strategy

Potential Cas9 cleavage sites were identified with the CRISPOR online tool,^50^ targeting the 5’ region of the 3’UTR, immediately downstream from the stop codon of the *scn10a* gene.

We chose sgRNAs binding very close to our targeted region, as the top 10 potential off-target sequences were not on the same chromosome (Chr 9) and could therefore be eliminated by segregation in successive backcrosses. In addition, the off-target hits on chromosome 9 displayed at least three mismatches, mostly at the 5’ and 3’ ends, which have been reported to be more important for target specificity.

sgRNA23: ACACTCCAGCATGCACGGGG GCC

sgRNA24: CACTCCAGCATGCACGGGGC CCC

##### *In vitro* validation of sgRNAs

For validation of the sgRNAs, the T7-sgRNA was amplified by PCR from the PX459 vector with the Phusion High-Fidelity DNA Polymerase (Thermo Fisher Scientific #F-530S), purified with Nucleospin Gel and the PCR-cleanup kit (Macherey Nagel #740609), validated by full sequencing (Eurofins Genomics) and used as a template for *in vitro* transcription with the Megashortscript T7 transcription kit (Life Technologies #AM1354). The sgRNAs were purified with the Megaclear kit (Life Technologies #AM1908), and an aliquot was subjected to electrophoresis in an agarose gel to check the integrity of the RNA. We assessed sgRNA efficacy with the Guide-it sgRNA screening system (Takara #632639). The DNA template for the sgRNA test *in vitro* was amplified from CK35 mouse gDNA by PCR with the Phusion High-Fidelity DNA polymerase and gel-purified.

All the primers used were obtained from Eurofins Genomics, and the pSpCas9(BB)-2A-Puro (PX459) V2.0 vector was a gift from Feng Zhang (Addgene plasmid #62988).

##### Generation of the sgRNA-Cas9 vector

The PX459 vector was digested with the BbsI restriction enzyme (New England Biolabs # R3539S), and gel-purified. Oligonucleotides for sgRNA23 were annealed and ligated to the linearized vector with the Zhang laboratory “Target Sequence Cloning protocol”. The ligation reaction mixture was used for the heat shock-mediated transformation of DH5a competent bacteria generated in-house. The clones obtained were tested by PCR and their DNA was sequenced. The final plasmid was purified with the Nucleobond Xtra Midi EndoFree kit (Macherey Nagel ref. 740420) for the electroporation of CK35 ES cells.

##### Generation of the targeting vector for homologous recombination

The targeting vector was assembled by standard cloning methods and did not contain guide and PAM sequences.

All fragments were amplified by PCR with the Phusion High-Fidelity DNA polymerase. The 5’ and 3’ homology arms, Ires2 and FLPo cassettes were amplified from CK35 ES cell gDNA, Ires2-pGEMTeasy and FLPo-pGK vectors, respectively, purified with NucleoSpin Gel and the PCR Cleanup kit and subcloned with the InFusion cloning kit (Takara ref. 639678). At various stages, the vectors were linearized with SacI or AatII restriction enzymes (New England Biolabs #R3156S and R0117S) and purified with NucleoSpin Gel and the PCR Cleanup kit before subcloning. The intermediate and final vectors were extracted with the Nucleospin Plasmid Mini-kit (Macherey-Nagel ref 740588) for sequencing.

Briefly, the 3’ arm was inserted into a SacI-linearized loxP-pGK-Neomycin-polyA-lox-pGEMTeasy vector, downstream from the pGK-Neo-pA cassette. In a second step, the 5’arm, Ires2 and FLPo cassettes were simultaneously inserted into the AatII-linearized first-step vector, upstream from the pGK-Neo-pA cassette. For both steps, clones were selected by PCR, DNA was extracted and the cloned fragments were fully sequenced (Eurofins Genomics).

After sequencing, the final targeting vector was purified with the Nucleobond Xtra Midi EndoFree kit and linearized with the AatII restriction enzyme for the electroporation of CK35 ES cells.

##### Generation and identification of founders

Targeting and sgRNA-Cas9 vectors were used to electroporate CK35 ES cells at the SEAT-CNRS facility (Villejuif-France). The targeted ES clones were identified by PCR and were then injected into blastocysts at the SEAT-CNRS facility. Founders were identified by genotyping (primers listed below) and backcrossed with C57BL/6J mice for five generations before intersection genetics crosses with the other mouse lines (Charles River Laboratory).

##### Genotyping primers:

WT-F: CAAATGACAACGGTGGGCTC

WT-R: TAATGGCCGACCCTCAGGTA

Mut-R (in Ires cassette): CAAAGGGTCGCTACAGACGTT

##### Screening for off-target hits

Potential off-target hits were identified with the CRISPOR tool. We analyzed only the off-target sites located on chromosome 9. We amplified 800 bp to 1 kb template fragments by PCR from the gDNA of G1 and G2 males for each selected off-target sequence and analyzed them with the EnGen® Mutation Detection kit (New England Biolabs #E3321). None of these fragments presented an digestions not displayed by the positive control from the kit. We also generated F4 homozygous mice, from which we amplified the same genomic DNA fragment, and sequenced it (Eurofins Genomics). All fragments had the expected WT sequence (C57BL/6J).

##### Tissue processing for immunofluorescence (IF) and *in situ* hybridization (ISH)

Mice were deeply anesthetized with 100 mg/kg ketamine plus 10 mg/kg xylazine. For cFos staining in the lumbar segment of the spinal cord, mice were anesthetized 1 h after CNO injection.

Mice were intracardially perfused with an ice-cold solution of phosphate-buffered saline (PBS) followed by 30 ml ice-cold 4% paraformaldehyde in PBS. Their tissues were dissected and post-fixed by overnight incubation in the same fixative at 4°C.

DRG, JNG, TG, glabrous hindpaw skin, heart, bladder, jejunum and liver tissues were transferred to 30% (w/v) sucrose in PBS for cryoprotection and incubated at 4°C until they sank. They were then frozen in OCT medium and stored at −80 °C. Samples with a thickness of 12 μm (DRG and JNG), 14 μm (TG sections), 16 μm (jejunum), 18 μm (heart and liver), 20 μm (glabrous skin) or 30 μm (bladder) were cut with a standard cryostat (Leica). All these tissue sections were mounted on Superfrost slides and kept at −80°C until their use for IHC experiments.

Brain and the lumbar segment of the spinal cord were mounted in a small 3% agarose block. Sections with a thickness of 80 μm (brain) or 50 μm (spinal cord) were cut with a Leica VT1200S vibratome. These sections were collected in a six- (brain) or 48 (spinal cord)-well plate filled with PBS and stored at 4°C until their use for IHC experiments.

##### Immunofluorescence

All our Immunostainings were performed in the lumbar region of the spinal cord (L3 to L5). The section shown in Figure 4E was chosen to highlight the spinal region that is densely innervated by the sensory fibers from the glabrous skin of the hind paw. For immunostaining, sections were incubated for 1 h at room temperature in PBS-10% (vol/vol) donkey serum (Sigma), 3% (weight/vol) bovine albumin (Sigma), 0.4% Triton X-100 and then overnight at 4°C with primary antibodies diluted in the same blocking solution. The primary antibodies used in this study were chicken anti-GFP (1:1000, Thermo Fisher Scientific, A10262); rat anti-RFP (1:1000, Chromotek, 5F8); rabbit anti-P2X3 (1:1000 Neuromics Cat# RA10109, RRID:AB_2157931); rat anti-GINIP (1:1000, Gaillard et al. 2014); rabbit anti-CGRP (1:1000, Cabiochem, PC205L, for DRG staining); rabbit anti-TH (1:500, Sigma-Aldrich AB152); rat anti-TAFA4 (1:2000, a gift from Sophie Ugolini (CIML)); rabbit anti-neurofilament M (145 kDa) (1:1000, Sigma-Aldrich AB1987); goat anti-TRKC (1:500, R and D Systems Cat# AF1404, RRID:AB_2155412); goat anti-CGRP (1:500, OriGene, BP022, for spinal cord staining); guinea pig anti-**VGLUT3** (1:1000, Synaptic Systems, 135204); rabbit anti-PGP9.5 (1:1000, Thermo Fisher Scientific, Cat# PA5-29012, RRID: AB_2546488), and rabbit anti-C-cFos (1:1000, catalog #ABE457, Millipore; RRID:AB_2631318). After three washes for 5 minutes each in 1xPBS, sections were incubated for 1 h at room temperature with secondary antibodies diluted in the blocking solution described above. The corresponding donkey anti-chicken, anti-rat, anti-rabbit, anti-goat or anti-guineapig Alexa 488-, 555-, or 647-conjugated secondary antibodies (1:500, Thermo Fisher Scientific) were used for the detection of primary antibody binding. Isolectin B4 conjugates with AlexaFluorR 647 dye were used at a dilution of 1:200 (Thermo Fisher Scientific I32450). Tissues were washed (3 times in 1xPBS) and mounted in ImmuMount Reagent. Images were acquired with an AxioImager M2 (Zeiss) fluorescence microscope with a 20x/0,8 objective (or a 10x objective for spinal cord in Figure 4D) and contrast was adjusted with Fiji software.

##### *In situ* hybridization

RNA probes were synthesized with gene-specific PCR primers and cDNA templates from mouse DRG. *In situ* hybridization was performed with digoxigenin-labeled probes (Roche, cat# 11277073910). Probes were incubated with the slides overnight at 55°C and the slides were then incubated with the horseradish peroxidase-conjugated anti-digoxigenin antibody 1:500 (Roche, Cat#11207733910; RRID:AB_514500). Final detection was achieved with TSA-Cy3 at a dilution of 1:50 (Perkin Elmer Life Sciences, FP1170). The oligonucleotides used for the nested PCRs and for probe synthesis are listed in the key resources table.

##### Cell counts

For ISH experiments, a threshold was set and scn10A-positive cells were then classified as Na_v_1.8 high or Na_v_1.8 low according to this threshold. The total number of cells analyzed per animal and per marker (scn10A and GFP) ranged from 571 to 1506.

For Figure S1D, the autofluorescence of the neurons made it possible to determine the total number of neurons. The total number of cells analyzed per animal and per genotype ranged from 962 to 4432.

##### Electrophysiology recordings

Dorsal root ganglion (DRG) neurons were prepared as previously described.^23^ Briefly, adult male C57BL/6J mice were anesthetized by pentobarbital injection and transcardially perfused with HBSS (pH 7.4, 4°C). Lumbar DRGs with attached roots were dissected out and collected in cold HBSS supplemented with 5 mM HEPES, 10 mM D-glucose and 1% penicillin/streptomycin. DRGs were treated with 2 mg/ml collagenase II and 5 mg/ml dispase for 40 min at 37°C, washed in HBSS and resuspended in 1 ml neurobasal A medium supplemented with B27, 2 mM L-glutamine and 1% penicillin/streptomycin (Invitrogen, Thermo Fisher Scientific, France). Single-cell suspensions were obtained by five passages of the cell suspension through three needle tips of decreasing diameter (gauge 18, 21, and 26). The cells were then plated on polyornithine/laminin-coated dishes. After incubation for 2 hours, the medium was removed and replaced with neurobasal B27 supplemented with 12.5 ng/ml NGF. Patch-clamp recordings were performed 12–24 h after plating, on GFP-positive neurons of 20 to 25 μM in diameter.

Macroscopic currents were recorded at room temperature with an Axopatch 200B amplifier (Molecular Devices, Sunnyvale CA). Borosilicate glass pipettes had a resistance of 1.5–2.5 MOhm when filled with an internal solution containing 100 mM CsCl, 40 mM TEA-Cl, 10 mM EGTA, 10 mM HEPES, 3 mM Mg-ATP, 0.6 mM GTP-Na, and 3 mM CaCl_2_ (pH adjusted to 7.25 with TEA-OH, ∼300 mOsm, ∼100 nM free Ca^2+^ according to Max-Chelator software, http://maxchelator.stanford.edu/). The extracellular solution contained 35 mM NaCl, 100 mM TEACl, 5 mM MgCl_2_, 0.1 mM CaCl_2_, 0.1 mM CdCl_2_, 5 mM KCl, 10 mM glucose and 10 mM HEPES (pH adjusted to 7.25 with TEA-OH, ∼300 mOsm). This extracellular solution also contained freshly prepared TTX (300 nM, Latoxan). Currents were elicited at 5 s intervals from a holding potential of -40 mV by test pulses of 30 ms duration from -40 mV to +50 mV with 5 mV increments. Recordings were filtered at 5 kHz. Data were analyzed with pCLAMP9 (Molecular Devices) and GraphPad Prism (GraphPad) software. Results are presented as the mean ± SEM, and *n* is the number of cells. Statistical analysis was performed with Student’s *t*-test.

##### Preparation of primary sensory neuron cultures for calcium imaging

All DRGs were carefully extracted and digested with 2mg/mL of collagenase type II (GIBCO), 5mg/mL of dispase (GIBCO) and 5mM of CaCl2 (Honeywell) in HBSS media (magnesium- and calcium- free) containing 5mM HEPES, 10mM D-Glucose and 1% penicillin/streptomycin, at pH 7.5 for 1h at 37°C. After removing the supernatant, DRGs were resuspended with Leibovitz-15 complete medium (L-15 supplemented with 5% FCS and 1% penicillin/streptomycin), and then mechanically dissociated with two glass Pasteur pipettes of decreasing diameter (1 and 0.5 mm). The resulting suspensions were subjected to a density gradient centrifugation through Percoll (onto 12.5 and 28% Percoll gradient in Leibovitz-15 complete medium) at a speed of 1300g (acc=6, break=3) for 20 minutes, to eliminate cell debris. Cells were washed with Leibovitz-15 complete medium and speed at 900g (acc=7, break=8) for 5 minutes. Cells were then resuspended in Neurobasal complete medium (Neurobasal A medium supplemented with 2mM L-glutamine, 1% penicillin/streptomycin and B27 1X) containing the following factors: Nerve growth factor (NGF) at 50ng/mL, Glial cell-derived neurotrophic factor (GDNF) at 50ng/mL, Neurotrophin-3 (NT3) at 50ng/mL, Neurotrophin-4 (NT4) at 10ng/mL and Brain Derived Neurotrophic Factor (BDNF) at 5ng/mL. Cells were plated in a Poly-D-lysine- (50 μg/ml) Laminin- (10 μg/ml) coated ibidi Polymer Coverslip Bottom and incubated at 37°C for 24 hours.

##### Calcium imaging

Calcium imaging (Ca^2+^) experiments were performed with an inverted microscope (Olympus IX73, 20X objective with a numerical aperture of 0.45) equipped with a LED illuminator (pE-300 white). Images were acquired at 10 Hz with an exposure time of 80 ms, with trueform waveform generators (33600B), and were recorded with a Basler acA4096 camera. Primary neuronal cultures were performed as described above and were stimulated with clozapine N-oxide (CNO) (25 μM), capsaicin (3 μM) and KCl (140 mM) solutions. They were then washed with Na-HEPES solution (containing 0.37 mg/ml KCl, 8.1 mg/ml NaCl, 2.38 mg/ml HEPES, 0.22 mg/ml CaCl_2_ and 0.19 mg/ml MgCl_2_, pH 7.3). All solutions were applied with syringes controlled by a Peri-Star Pro pump. Calcium responses were recorded before, during and after stimulation. Before each experiment, DRG neurons were incubated with 400 μl Opti-MEM solution and 5 μM calcium dye (Fluo4-AM). Cells were imaged with Pylon Viewer software. All experiments were performed at room temperature (25°C). The data were analyzed with an in-house Matlab code (Matlab 2018A). Data are presented as the relative change in fluorescence (ΔF/F0) of each neuron, where F0 is basal fluorescence and ΔF = F − F0. All cells that did not respond to KCl or capsaicin were excluded from the analysis.

Regardless of their response to CNO, the cells retained for the final analysis were those that responded to capsaicin alone, KCl alone or to both compounds.

##### CNO preparation

CNO (6329, TOCRIS, batch no.: 4A/271069) was dissolved in water at a concentration of 10 mg/ml (w/v) and then aliquoted and stored at -20°C. CNO solution was freshly prepared at a concentration of 25.5 μM (for *in vitro* experiments) or 2 mg/ml (for *in vivo* experiments) by diluting the 10 mg/ml stock solution with Na-HEPES washing solution (for *in vitro* experiments) or 0.9% NaCl (for *in vivo* experiments).

##### Behavioral test

Behavioral assays were conducted on 8- to 12-week-old Na_v_1.8^FLPo/+^::Advilin^+/+^ ::R26^RC-FL-hM3Dq/+^ (Cre^-^, control mice) and Na_v_1.8^FLPo/+^::Advilin^Cre/+^ ::R26^RC-FL-hM3Dq/+^ (Cre^+^) mice. Animals were allowed to get used to the experimenter before the tests were performed. Mice were placed individually into Perspex chambers and were allowed to acclimate to the testing environment for 30 min. We then injected 10 μl of a 2 mg/mL CNO solution subcutaneously into the plantar surface of the left hind paw. The animals were immediately placed in observation chambers and monitored for pain-related behavior (shaking, licking and biting of the injected paw) for 30 min. The cumulative duration of pain-related behavior was determined in seconds, at five-minute intervals. The experimenter was blind to the genotype of the mice.

### Quantification and statistical analysis

The number of animals tested is indicated in the figure legends. The Shapiro–Wilk test was used to assess data normality. Values of *p* < 0.05 were considered to be statistically significant in unpaired *t*-tests for IF experiments or unpaired Mann-Whitney tests for *in vivo* experiments. Statistical analyses were performed with GraphPad Prism 9.0 (GraphPad Software, Inc., San Diego, CA).

## Supplemental information

Document S1. Figures S1 and S2

Video S1. Video of a Cre-mouse behavior during the first minute following CNO intraplantar injection, related to Figure 4 CNO intraplantar injection does not result in spontaneous pain behavior.

Video S2. Video of a Cre+ mouse behavior during the first minute following CNO intraplantar injection, related to Figure 4 CNO intraplantar injection results in lifting, licking and biting responses of the injected paw.

Video S3. Video (speedX0.25) of an example spontaneous CNO-induced pain behavior quantified during the test, related to Figure 4 CNO intraplantar injection results in a robust shaking and stretching responses of the injected paw. A mouse showing this type of behavior repeatedly over the test period corresponds to the highest experimental points on the graph in figure 4.
