# Supplementary material for: A novel Nav1.8-FLPo driver mouse for intersectional genetics to uncover the functional significance of primary sensory neuron diversity
Source: iScience. 2024 Mar 5;27(4):109396. doi: 10.1016/j.isci.2024.109396 (PMC10952036; doi:10.1016/j.isci.2024.109396)
Supplement: Document S1. Figures S1 and S2 [file mmc1.pdf]

**iScience, Volume *27***

## Supplemental information

# A novel $Na_v$1.8-FLPo driver mouse for intersectional genetics to uncover the functional significance of primary sensory neuron diversity

**Pascale Malapert, Guillaume Robert, Elena Brunet, Jean Chemin, Emmanuel Bourinet, and Aziz Moqrich**

## Supplemental information

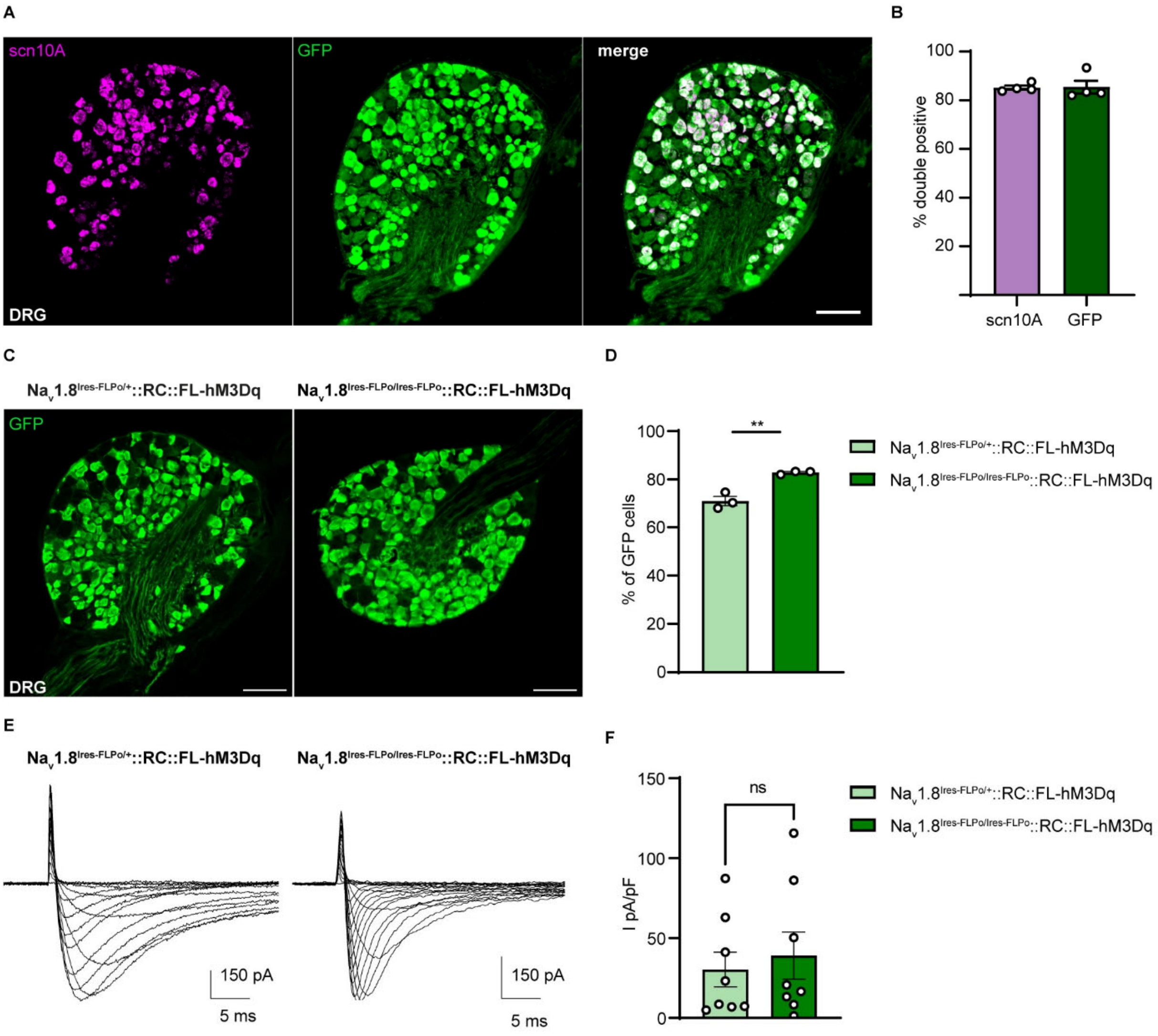


**Figure S1_Deeper characterization of DRG sensory neurons expressing the functional FLPo in the Nav1.8-Ires-Flpo mouse model, related to Figure 1**

(A) In-situ hybridization on thoracic DRG section using antisense probe for *scn10A* gene (magenta) followed by immunostaining using chicken anti-GFP (green), from a Nav1.8$^{Ires-FLPo/+}$::R26$^{hM3Dq/+}$. Scale bar: 100 µm.

(B) Quantification of the percent of double positive cells from within the *scn10A* positive population or double positive cells within the GFP positive population in thoracic DRG sections

from a Nav1.8$^{Ires\text{-}FLPo/+}$::R26$^{hM3Dq/+}$ (N=4 mice, individual means from each shown as a single dot. Bars represent mean value from all four animals, data are presented as mean ± SEM).

(C) GFP immunostainning on thoracic DRG sections from a Nav1.8$^{Ires\text{-}FLPo/+}$::R26$^{hM3Dq/+}$ mouse (left) and from a Nav1.8$^{Ires\text{-}FLPo/Ires\text{-}FLPo}$::R26$^{hM3Dq/+}$ mouse (right). Scale bar: 100 µm.

(D) Quantification of the percent of GFP positive cells from within the total number of DRG neurons. (N=3 mice for each genotype, individual means from each shown as a single dot. Bars represent mean value from all three animals, data are presented as mean ± SEM (unpaired t-test, **p =0.0039).

(E) TTX-resistant Na-currents recorded in DRG neurons obtained from Nav1.8$^{Ires\text{-}FLPo/+}$::R26$^{hM3Dq/+}$ and Nav1.8$^{Ires\text{-}FLPo/Ires\text{-}FLPo}$::R26$^{hM3Dq/+}$ mice. Currents were elicited by a 30 ms depolarization at -40 to +50 mV (5 mV increment) from a holding potential of -40 Mv.

(F) Maximal TTX-resistant Na-current density (pA/pF) obtained from Nav1.8$^{Ires\text{-}FLPo/+}$::R26$^{hM3Dq/+}$ and Nav1.8$^{Ires\text{-}FLPo/Ires\text{-}FLPo}$::R26$^{hM3Dq/+}$ mice.

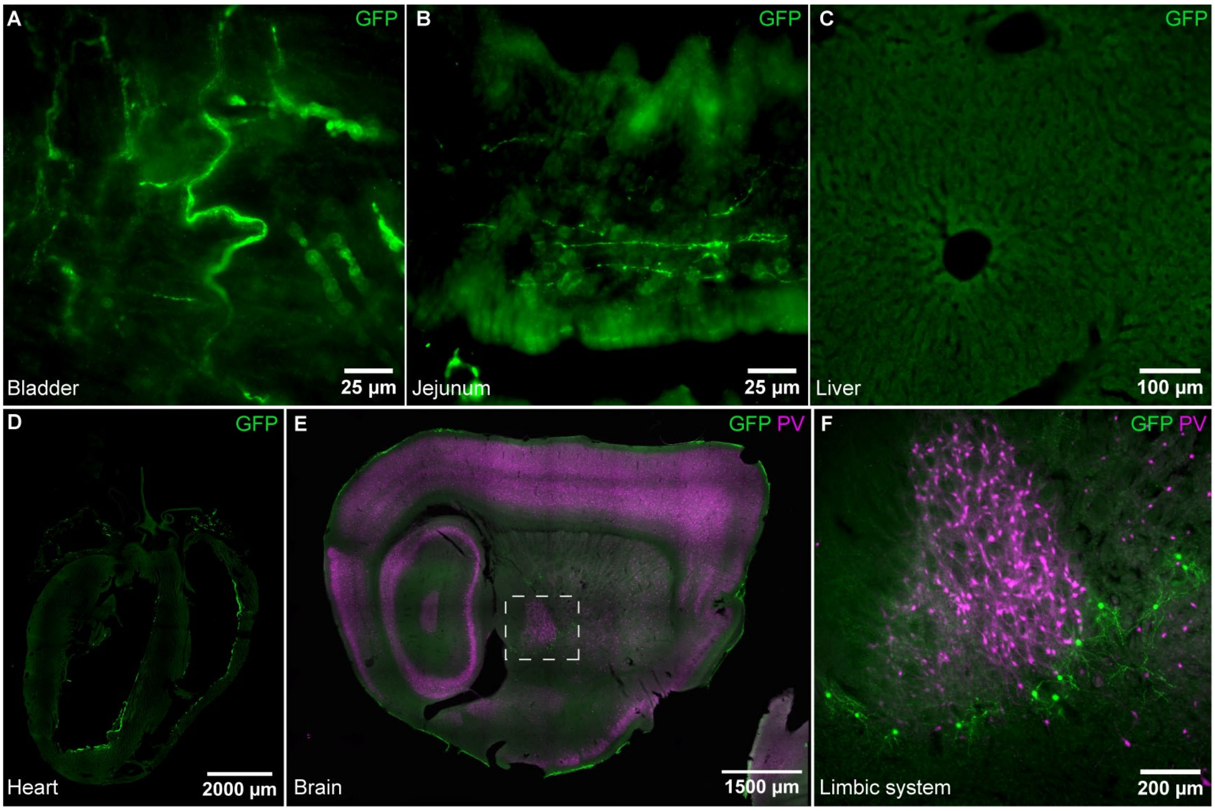


**Figure S2_Deeper characterization of the functional Flippase expression in the $Na_v1.8$-FLPo mouse model, related to Figure 2**

(A-E) Tissue sections immunolabelled with GFP (green) from a $Na_v1.8^{Flpo/+}$::$R26^{hM3Dq/+}$ mouse: (A) Bladder, (B) Jejunum, (C) Liver,and (D) Heart.

(E) whole brain sagittal section co-immmunolabelled with GFP (green) and PV (magenta) from a $Na_v1.8^{Flpo/+}$::$R26^{hM3Dq/+}$ mouse. (F) Zoom-in view of the selected areas in (E)

(F) Limbic system region co-immmunolabelled with GFP (green) and PV (magenta) from a $Na_v1.8^{Flpo/+}$::$R26^{hM3Dq/+}$ mouse.
